# Analysis of significant factors for dengue fever incidence prediction

**DOI:** 10.1186/s12859-016-1034-5

**Published:** 2016-04-16

**Authors:** Padet Siriyasatien, Atchara Phumee, Phatsavee Ongruk, Katechan Jampachaisri, Kraisak Kesorn

**Affiliations:** Department of Parasitology, Faculty of Medicine, Chulalongkorn University, Bangkok, 10330 Thailand; Excellence Center for Emerging Infectious Disease, King Chulalongkorn Memorial Hospital, Thai Red Cross Society, Bangkok, 10330 Thailand; Department of Computer Science and Information Technology, Faculty of Science, Naresuan University, Phitsanulok, 65000 Thailand; Department of Mathematics, Faculty of Science, Naresuan University, Phitsanulok, 65000 Thailand

**Keywords:** Dengue hemorrhagic fever, Forecasting model, Prediction model, Multivariate poisson regression, Climate factor analysis

## Abstract

**Background:**

Many popular dengue forecasting techniques have been used by several researchers to extrapolate dengue incidence rates, including the K-H model, support vector machines (SVM), and artificial neural networks (ANN). The time series analysis methodology, particularly ARIMA and SARIMA, has been increasingly applied to the field of epidemiological research for dengue fever, dengue hemorrhagic fever, and other infectious diseases. The main drawback of these methods is that they do not consider other variables that are associated with the dependent variable. Additionally, new factors correlated to the disease are needed to enhance the prediction accuracy of the model when it is applied to areas of similar climates, where weather factors such as temperature, total rainfall, and humidity are not substantially different. Such drawbacks may consequently lower the predictive power for the outbreak.

**Results:**

The predictive power of the forecasting model-assessed by Akaike’s information criterion (AIC), Bayesian information criterion (BIC), and the mean absolute percentage error (MAPE)-is improved by including the new parameters for dengue outbreak prediction. This study’s selected model outperforms all three other competing models with the lowest AIC, the lowest BIC, and a small MAPE value. The exclusive use of climate factors from similar locations decreases a model’s prediction power. The multivariate Poisson regression, however, effectively forecasts even when climate variables are slightly different. Female mosquitoes and seasons were strongly correlated with dengue cases. Therefore, the dengue incidence trends provided by this model will assist the optimization of dengue prevention.

**Conclusions:**

The present work demonstrates the important roles of female mosquito infection rates from the previous season and climate factors (represented as seasons) in dengue outbreaks. Incorporating these two factors in the model significantly improves the predictive power of dengue hemorrhagic fever forecasting models, as confirmed by AIC, BIC, and MAPE.

## Background

Incidences of dengue hemorrhagic fever (DHF) and dengue fever (DF) have increased dramatically in past decades and have become a global threat. According to the World Health Organization (WHO), an estimated 500 million cases of DF and 250,000–500,000 cases of DHF occur annually [1, 2]. The number of people residing in at-risk areas of DF outbreak totals 3.6 billion, or 55 % of the world’s population [3]. Thailand recorded its first case of DF in 1958 [4]. Since then, the disease has become a major public health problem as the number of cases has continued to expand. Dengue virus infection can cause dengue diseases including classical DF and its severe form, namely, DHF and/or dengue shock syndrome (DSS) [5]. Approximately 10–15 % of infected patients are symptomatic, with ~500,000 hospitalizations annually involving the severe form of the disease [6]. The annual hospitalization and death rates of patients by the severe form is highest in tropical and subtropical regions, especially in Southeast Asia, South and Central America, the Caribbean and South Pacific [5]. Huge efforts to control and monitor the dengue epidemic are currently underway in many countries. The major vector of dengue is the mosquito *Aedes aegypti* [4]. Females of this species transmit the virus to humans when taking a blood meal. Early warning system of dengue outbreak and advising the relevant departments to deploy mosquito control prior to disease outbreak is essential. Barbazan et al. [7] demonstrated that the seasonal transmission of DENV serotypes in an endemic area was significantly related to the prevalence and virulent strains and also associated to the high pathogenesis. Some studies suggested that active school-based dengue detection could be used as an indicator for reducing the longitudinal risk of viral transmission in rural areas [8].

Although the disease is transmitted to humans via female mosquitoes, entomologic surveillance to determine dengue transmission has been based on different larval indices [9, 10] including house index (percentage of houses positive for larvae) and the Breteau index (number of positive containers per 100 houses). Even though, these indices have become widely used for dengue control program, prevalence of dengue infection is still high especially in the rainy season. With the advent of molecular biology techniques, it was possible to detect dengue viruses in mosquito vectors [11]. The virus infection in mosquito was then considered as an index to determine dengue epidemic. Several reports demonstrated the relationship between dengue outbreak and virus infection in *Ae. aegypti* mosquitoes. This correlation seems to be more practical and effective tool for planning dengue control [12–16]. Nonetheless, dengue incidence is difficult to predict because it varies widely over time [17]. Many DF prediction models are based on statistical and data mining techniques such as ARIMA [18], SARIMA [19–21], the K–H model [17], support vector machines (SVMs) [22], and artificial neural networks (ANNs) [23]. All of these approaches adopt a similar basic set of predictors, such as temperature and rainfall level. To enhance the predictive power of DF models, we incorporated two novel predictors, female mosquito infection rate and season.

## Methods

### Study site for mosquito collection

**Fig. 1:**
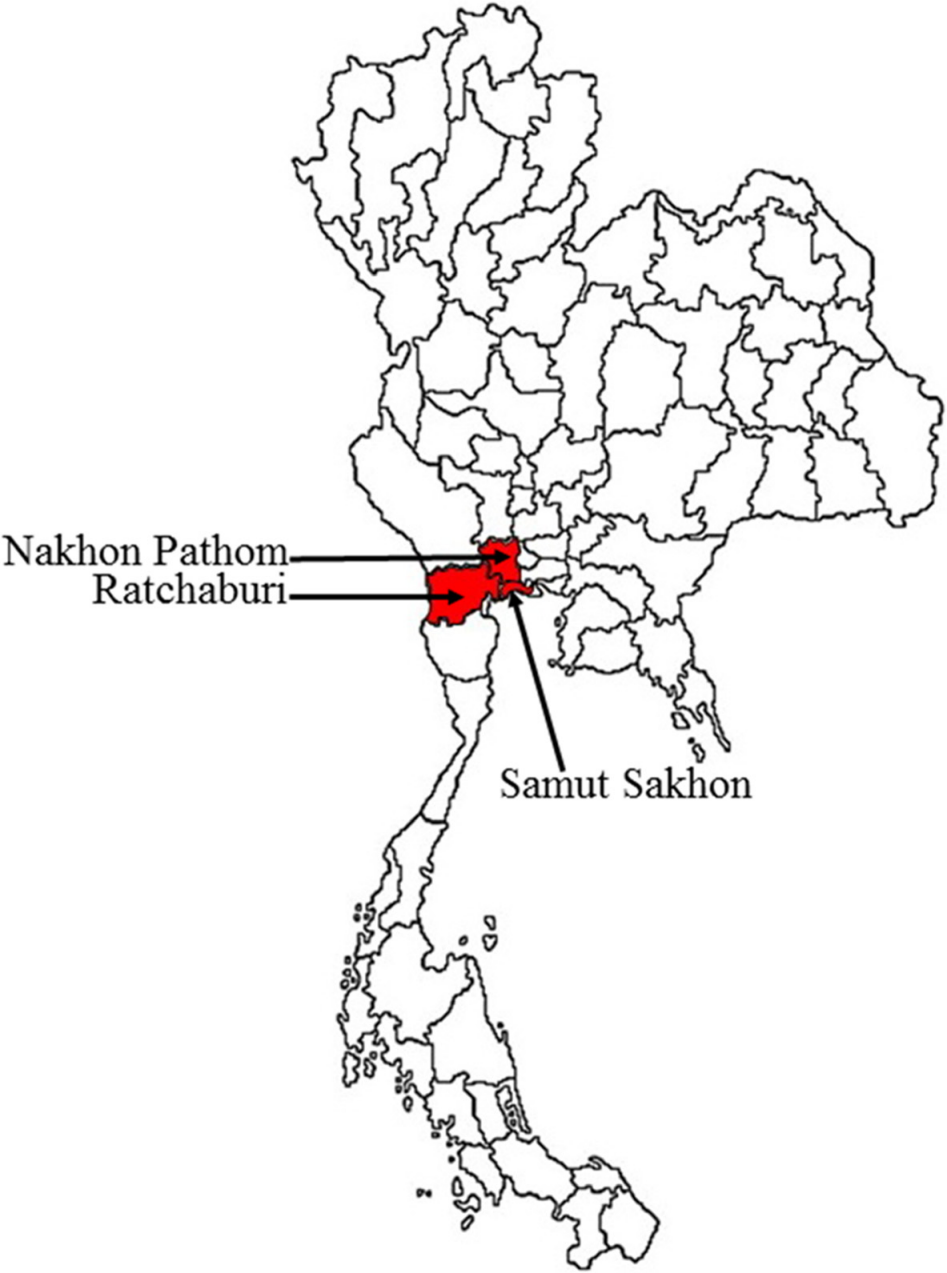
Map of morbidity rate of dengue in Thailand reported by Health Info in Thailand (http://www.healthinfo.in.th/). The study areas were the three provinces of Nakhon Pathom, Ratchaburi, and Samut Sakhon in the central region of Thailand. The high morbidity rate (per 100,000 populations) of DHF between 2007 and 2012 is indicated by the *red color*

From 2007 to 2012, *Ae. aegypti* mosquitoes were collected from three provinces in the central region of Thailand, including Nakhon Pathom, Ratchaburi, and Samut Sakhon. These areas were selected primarily for three reasons: high mosquito density, minor differences in climatic factors, and a high DHF morbidity rate as reported in Thailand health information system (http://www.hiso.or.th) and as illustrated in Fig. 1.

### Ethics statement

The study was approved by the Ethics Committee of Research Affairs Unit, Faculty of Medicine, Chulalongkorn University (COA No. 328/2014).

### Dengue mosquito collection

*Ae. aegypti* larvae and adults mosquitoes were collected from three provinces in the central region of Thailand. The collections were performed in three districts of each province (two sub-districts per district; two villages per sub-district; 40 dwellings per village). Twice per season, from January 2007 to December 2012, mosquito larvae were collected from water-filled containers indoors and around the houses; adult mosquitoes were collected by highly experienced officers from Thailand’s National Institute of Health using human bait. Larvae and adults were visually identified as members of *Ae. aegypti* and were pooled, then maintained, in cryogenic vials. Each vial contained five larvae or mosquitoes and was stored in liquid nitrogen for subsequent dengue virus detection. Dengue virus infected mosquito rates were obtained from a previous report by Chompoosri et al. [14].

### Dengue virus detection in Ae. aegypti mosquitoes

Detection of the four dengue virus serotypes in *Ae. aegypti* larvae and adults was modified from the method described by Tuksinvaracharn et al. [11]. The genomic viral RNA was extracted from pooled larvae and mosquitoes using the Invisorb® Spin Virus RNA Mini Kit (Invitex Gmbh, Germany) according to the manufacturer’s protocols. One-step RT-PCR was performed with five oligonucleotide primers (D1 and four type-specific primers, including TS1, TS2, TS3, and TS4) that were designed by Lanciotti et al [24]. Amplification was carried out in a 25 μl total mixture using the Superscript III one-step RT-PCR kit (Invitrogen, USA) with 10 μM of each primer and 6 μl of RNA. The RT-PCRs were performed in a PCR Mastercycler® Pro (Eppendorf, Germany) under the conditions of 50 °C for 30 min and 94 °C for 2 min, followed by 40 cycles of 94 °C for 30 s, 50 °C for 30 s, and 72 °C for 30 s; finally, the last cycle was at 72 °C for 7 min followed by a final holding at 4 °C. Aliquots of the PCR amplicons were analyzed by electrophoresis on 2 % agarose gels, stained with ethidium bromide, and visualized with Quantity One Quantification Analysis Software version 4.5.2 (Gel Doc EQ System; Bio-Rad, Hercules, CA).

### Incidence of DHF in the study areas and dengue virus detection in blood samples

Incidences of DHF in the study areas were obtained from the Bureau of Epidemiology, Department of Disease Control, Ministry of Public Health, Thailand. The data were expressed as the morbidity rate of DHF per 100,000 individuals. Blood specimens were taken from suspected dengue infection patients, with 3 ml of blood collected into EDTA collecting tubes from each patient. Identification of dengue serotypes was performed by one-step RT-PCR [25]. Viral RNA was extracted from 100 μl of plasma from each patient, and RT-PCR for type-specific primers was carried out using a one-step RT-PCR kit (Qiagen Gmbh, Hilden, Germany). Each amplification was validated with positive and negative controls. PCR products were electrophoresed in 2 % agarose gel, stained with ethidium bromide (0.5 μg/ml), and visualized on a UV transilluminator (Gel Doc EQ System; Bio-Rad, Hercules, CA). The study was approved by the Ethics Committee of Research Affairs Unit, Faculty of Medicine, Chulalongkorn University (COA No. 328/2014).

### Independent and dependent variables for a forecasting model

Besides the abovementioned mosquito infection rate parameters, data for all other factors relevant to DHF outbreaks were collected from various sources. Table 1 lists the independent and dependent variables considered in the proposed forecasting model. Values of all variables were collected between 2007 and 2012. Mosquito and blood sample collections were performed only until 2012 owing to budget limitations.

**Table 1:** Independent and dependent variables used in the proposed forecasting model

Independent variables	Source	Data type	Unit
1. Average temperature (AvgTemp)	Thai Meteorological department	Continuous	Celsius (°C)
2. Average rainfall (AvgRain)	Thai Meteorological department	Continuous	Millimeters (mm)
3. Average humidity (AvgHumid)	Thai Meteorological department	Continuous	Percentage (%)
4. Average wind speed (AvgWind)	Thai Meteorological department	Continuous	Miles per hour (mph)
5. *Ae. aegypti* larvae infection rate (AegRate)	Parasitology Department, Chulalongkorn University	Continuous	Percentage (%)
6. Female mosquito infection rate (Fmosquito)	Parasitology Department, Chulalongkorn University	Continuous	Percentage (%)
7. Male mosquito infection rate (Mmosquito)	Parasitology Department, Chulalongkorn University	Continuous	Percentage (%)
8. Season	-	Nominal	N/A
9. Population (Pop)	Total population in each studied region	Continuous	Number of people
10. Dengue cases	National Trustworthy and Competent Authority Epidemiological Surveillance and Investigation Department (NTCAESI)	Continuous	Cases per 100,000 individuals

All collected data were cleaned before performing the analysis. Data cleansing transforms the data and removes those with missing values. After data cleansing, observations in each district were pooled seasonally; 144 samples remained and were used for model construction. Seasonal temperature, rainfall, humidity, and wind had indicated significantly high correlation coefficients (*p* < 0.0001) among themselves, as shown in Table 2-resulting in a multicollinearity problem in model fitting, and decreasing the reliability of the model. Therefore, we used the season variable as a proxy for meteorological conditions.

**Table 2:** Correlation coefficient of climate factors

	AvgRain	AvgTemp	AvgWind	AvgHumid
AvgRain	1.00	−0.66^*a*^	−0.40	0.64
		<0.0001^*^	<0.0001	<0.0001
AvgTemp		1.00	0.59	−0.97
			<0.0001	<0.0001
AvgWind			1.00	−0.59
				<0.0001
AvgHumid				1.00

^*a*^Pearson correlation coefficient

^*^ *p*-value

**Fig. 2:**
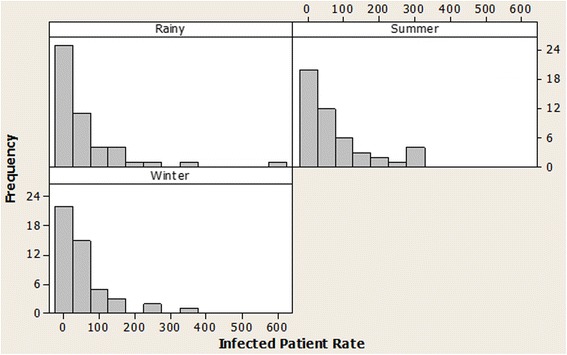
Histogram of dengue rate in the studied regions from 2007 to 2012, classified by season

Dengue rates in each season of the studied regions were explored, indicating right-skewed distribution, as illustrated in Fig. 2. Multivariate Poisson regression (MPR) [26], frequently applied to the analysis of count data [27] due to non-normal distribution, was adopted to find variables associated with the number of dengue cases; the main significant variables were initially selected for the model using the backward elimination scheme. Subsequently, two-variable interactions were added, and their effects were tested hierarchically. However, count data in the Poisson model usually displayed larger variation than its mean, referred to as “overdispersion.” Here, we accommodated the overdispersed model by adjusting the parameter covariance matrix and likelihood function, yielding a more appropriate standard error estimation and likelihood ratio test.

A previous study [12] revealed that dengue infection rates in female mosquitoes of three provinces were highest in summer, while morbidity rates of DHF tended to be highest in the rainy season. Consequently, female mosquito infection rate in the previous season (one lag season) is used in predicting the number of dengue infections. As depicted in Table 3, four main variables are first considered in the model fitting process.

**Table 3:** The effect of four main variables on dengue incidence in MPR fitting

Variables	LR^*a*^	*p*-value
Season	5.50	0.064
Fmosquito	3.53	0.060
Mmosquito	2.60	0.107
AegRate	1.14	0.285

^*a*^likelihood ratio statistics

### Model construction

#### Multivariate poisson regression

In our previous study [15], we showed the significance of the infected female mosquito but did not study the correlation among the climate factors. In this paper, we deploy the season variable instead of climate factors. Additionally, we proposed to exploit the MPR technique that accounts for multiple predictors. Retrospective data are collected on a seasonal basis and the model temporally extrapolates the dependent variable by several seasons. Typically, the regression model expresses the natural logarithm of outcome as a linear function of a set of predictors, as shown in Eq. 1.

1 $$ \ln \left({\mu}_i\right)={\beta}_0+{\beta}_1\ \mathrm{Season}{1}_i+{\beta}_2\ \mathrm{Season}{2}_i+{\displaystyle \sum_{j=3}^5{\beta}_j{X}_{ji}+ \ln \left(po{p}_i\right)} $$

where ln(*μ*_*i*_) is the natural logarithm of predicted seasonal dengue incidence of the *i*^th^ observation; ln(*pop*_*i*_) is the natural logarithm of population and used as an offset accounting for variation of population among regions; *β*_0_ is the constant, denoting the baseline number of dengue incidences; *β*_1_ and *β*_2_ are regression parameters, denoting the effect of Season1 (Rainy) and Season2 (Summer) compared with Season3 (Winter); and *β*_*j*_ ' *s* denote the effect of independent variables *X*_*j*_ on dengue incidence, representing Fmosquito, Mmosquito, and AegRate, where *j* = 3, 4, and 5, respectively.

Initially, four main variables were considered in the model fitting; variables were then removed one by one based on the backward elimination procedure. Two-factor interactions of the remaining variables were then added. The final model was ultimately selected based on three measures: the Akaike information criterion (AIC), the Bayesian information criterion (BIC), and the mean absolute percentage error (MAPE). All competing models were also compared in nested order for model selection.

#### Multivariate poisson regression model validation

The constructed model was evaluated by three performance measures; MAPE, AIC, and BIC. The MAPE is given by Eq. (2).

2 $$ MAPE=\frac{1}{n}{\displaystyle \sum_{i=1}^n\left|\frac{X_i-{F}_i}{X_i}\right|} $$

where *X*_*i*_ and *F*_*i*_ are the observed and predicted values, respectively, and *n* is the total number of observations. The AIC [28] and BIC [29], illustrated in Eqs. (3) and (4), were considered in model selection to assess the goodness-of-fit of the model.

3 $$ AIC=2k-2 \ln L $$

4 $$ BIC=-2 \ln L+k \ln n $$

where *k* is the number of model parameters, and *L* is the maximized value of the likelihood function for the model. Lower MAPE, AIC, and BIC values indicate increased predictive power.

## Results and discussion

The collected data from 2007 to 2012 were used for model construction. The number of dengue cases over time was then predicted based on the chosen model. Finally, the forecasted cases were compared with the actual dengue cases reported by NTCAESI. The dataset in this experiment includes all variables listed in Table 3 from the three provinces.

### Model selection

The best model (yielding the lowest MAPE, AIC, and BIC) was selected in subsequent experiments. Four MPR models were constructed, and their MAPEs, AICs, and BICs were compared. To account for climate effect on dengue cases, the categorical variable of season was included into the model fitting. The first model (Model-1) deployed all four main predictors, whereas the second model (Model-2) and third model (Model-3) excluded insignificant terms. AegRate and Mmosquito were highly correlated with Fmosquito (*r* = 0.61, *p* < 0.001; and *r* = 0.57, *p* < 0.001), implying that each conveyed a similar relationship to dengue cases as that of Fmosquito; both variables were thus removed from Model-3. Subsequently, the interaction of two newly identified main factors (Season and Fmosquito) was added into the fourth model (Model-4), according to the model selection process. The AIC, BIC, and MAPE values obtained from each model, including data from three provinces, are listed in Table 4.

**Table 4:** Model comparison

Model	Variables	AIC	BIC	MAPE (%)	Model compared	Chi-square	*p*-value
1	Season + Fmosquito + Mmosquito + AegRate	160.33	178.15	325.38	-	-	-
2	Season + Fmosquito + Mmosquito	159.32	174.17	336.96	2 vs. 1^*a*^	0.49	0.482
3	Season + Fmosquito	*158.27*	*170.15*	326.81	3 vs. 2^*b*^	0.47	0.491
4	Season + Fmosquito + Season × Fmosquito	160.15	177.96	*320.27*	3 vs. 4^*c*^	1.06	0.588

^*a*^H_0_: Model-2 is appropriate vs. H_1_: Model-1 is appropriate

^*b*^H_0_: Model-3 is appropriate vs. H_1_: Model-2 is appropriate

^*c*^H_0_: Model-3 is appropriate vs. H_1_: Model-4 is appropriate

When one model was a special case of another, models can be compared in hierarchical order whereas simpler model in the null hypothesis was tested against more complex model in the alternative hypothesis. When the hypothesis testing indicated insignificance, the simpler model was adequate and the model under the null hypothesis is supported. As shown in Table 4, Model-3 yielded the lowest AIC and BIC. Although Model-4 and Model-1 gave smaller values of MAPE than Model-3, the extra terms did not significantly affect dengue case prediction. This discovery was not surprising because models with more attributes usually provide greater prediction power. Additionally, models are traditionally compared under the null hypothesis that the simpler model with fewer terms is better-similar to the principle of parsimony [26]. As all of these assessments revealed Model-3 to be the best model, Model-3 has been adopted as a representative model for predicting dengue incidences throughout the remainder of this study.

### Multivariate poisson regression model analysis

Having selected a model, we quantitatively associated each variable with dengue cases. Table 5 lists the estimation of regression coefficients, standard errors, Wald statistics, and *p*-values of the selected model.

**Table 5:** Estimation of regression coefficients, standard errors, Wald statistics, and *p*-values of the MPR model

Variables^a^	Coefficient	Standard error	Wald statistics	*p*-value
Intercept	−8.16	0.28	833.17	<0.001
Season1	0.55	0.26	4.69	0.030
Season2	0.24	0.26	0.83	0.361
Fmosquito	0.02	0.01	5.90	0.052

^a^Season1 = rainy (May–Aug); Season2 = summer (Jan–Apr); Season3 = winter (Sep–Dec) and is the baseline of this analysis

Substituting the coefficients of Table 5 into Eq. 1, we obtain

5 $$ \ln \left({\mu}_t\right)=-8.16+0.55\ \mathrm{season}1+0.24\ \mathrm{season}2+0.02\ \mathrm{Fmosquito}+ \ln (pop) $$

In the next subsection, dengue incidences are forecasted by Eq. 5, and the predictions are compared with the actual data.

The regression coefficients in Table 5 indicate that a 1 % increase in the number of infected female mosquitoes from the previous season will generate a 1.02-fold (e^0.02^) increase in the number of dengue incidences. The spread of dengue may be explained by several factors. In addition to the transmission of dengue virus to humans from mosquito bites, viral transmission among mosquitoes may also occur through transovarian [14] transmission. When increasing numbers of mosquitoes are infected, there is also naturally an increased risk that people living in such mosquito-infested areas may contract the disease. Outbreak risk is highest during the rainy season (Season1), being 1.73 (e^0.55^) times higher than that of the winter season (baseline). The severity of the outbreak is raised by a factor of e^0.24^ = 1.27 during the seasonal changeover from winter to summer (Season2) and by a factor of e^0.55-0.24^ = 1.36 during the changeover from summer to the rainy season. This result is attributed to the large volumes of standing water on private properties that accumulate during the rainy season. Standing water revives mosquito eggs that have lain dormant over the previous seasons, with subsequent surges in mosquito emergence.

### Prediction performance

**Fig. 3:**
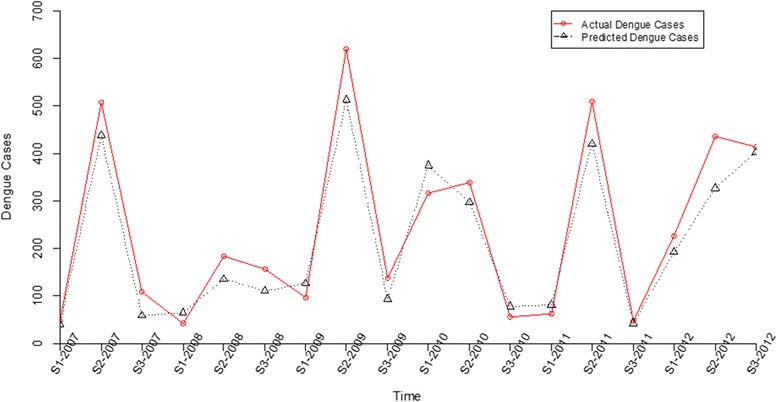
Line chart comparing actual dengue cases and predicted values between 2007 and 2012

Owing to high mobility rate in the rainy season, this study only demonstrates the prediction performance for this season. According to the results illustrated in Fig. 3, both actual and predicted value tended to demonstrate similar trends across the year, reflecting good performance of the adopted MPR model. In addition, Kolmogorov-Smirnov test [30] is utilized to verify the prediction performance and to test whether the actual and predicted value are consistent. The null hypothesis of consistency between actual and predicted value is not rejected, with D = 0.17 (*p*-value = 0.9639), indicating consistency of the actual and predicted values. Because our model accounts for the overdispersed problem, covariance estimation is adjusted to improve reliability. As a result, the prediction performance of the model is significantly improved.

## Conclusions

As mentioned previously, no specific treatment exists for dengue infection, and effective vaccines remain at the developmental stage. Therefore, interrupting pathogen transmission by mosquito control is the most effective means of controlling dengue infection. In Thailand, although mosquito surveillance has been in regular operation for many years, surveillance has not appeared to fully prevent dengue outbreaks. Seasonal factor has been previously studied by Wongkoon et al. [31] which is similar to the work in this report. Nonetheless, the main differences between these works are: firstly, we did not exploit only the number of *Ae. aegypti* populations found in urban and rural areas as demonstrated by Wongkoon et al. [31] but the dengue virus infection rate of larva and adult *Ae. aegypti* mosquitoes, which has never been determined for dengue fever transmission, was exploited. We incorporated the infected *Ae. aegypti* larvae, female and male mosquito and attributes together with seasonal variable into the predicted model in order to enhance the prediction power. This is because female mosquito can transmit dengue viruses through larvae by trans-ovarian transmission [12, 32, 33] and male mosquitoes can transmit the viruses to females via sexual transmission [12, 33]. We found that the infected female mosquito together with season are directly correlated to the number of dengue cases and significantly useful for the forecasting model as confirmed by the results shown in Fig. 3. Secondly, Wongkoon et al. [31] used the container, house, and Breteau indices to determine dengue transmission as similar to several other previous reports [34–36]. However those indices may not correlate to the dengue virus transmission due to the increasing of dengue cases in Thailand. Therefore, female mosquito and season could be used as novel variables for effectively determination of the dengue outbreak in Thailand.

Infected female mosquito has also been used to predict dengue cases in our previous work [15]. However, the prediction techniques of these works are different; data mining-based technique (SVM, Neural network, Decision tree, and K-nearest neighbor) were used in the previous work to construct the forecasting model whereas statistics-based technique (Multivariate Poisson regression) was used in this work. Statistics is well established methodology of science and useful for verifying relationships among parameters when the relationships are linear while data mining techniques are useful for knowledge finding hidden in the data. In this paper, we focus on the analysis of linear correlation between dengue cases and infected data of mosquito. As such, methodology for model analysis and selection are different and they are the major contribution in this paper.

The present work demonstrates the important roles of female mosquito infection rate and season in dengue outbreak prediction. Statistic-based analysis illustrated that there is a positive relation between these variables and the number of dengue cases. Hence, integrating these two factors in the forecast model significantly improves the model’s DHF predictive power, as confirmed by AIC, BIC, and MAPE. The proposed model efficiently estimated the dengue incidence trends in the trial experiments reported here and could assist in dengue outbreak surveillance and control at the early stages, before outbreaks spread. Although dengue virus infection rate in mosquito is effective for prediction of dengue outbreak, but the technique is costly and time consuming therefore it has never been used to determine dengue outbreak in previous reports. To date technique for rapid detection of dengue virus such as loop-mediated isothermal amplification (LAMP) is developed [37]. LAMP reactions can be observed by naked eyes [38] and the technique has low cost therefore it could be used to determine dengue virus infection rate in mosquito in the field survey. Dengue infection rate in mosquitoes could be incorporated in the dengue control measure in the near future. Currently, we are extending the model to other factors that could potentially enhance model performance. Landscape, dengue serotypes, and demographic transitions in the target areas are some of the additional factors now undergoing further investigation.

## References

[1] Beatty ME, Stone A, Fitzsimons DW, Hanna JN, Lam SK, Vong S (2010). Best practices in dengue surveillance: A report from the Asia-Pacific and Americas Dengue Prevention Boards. PLoS Negl Trop Dis.

[2] Faisal T, Taib MN, Ibrahim F (2012). Neural network diagnostic system for dengue patients risk classification. J Med Syst.

[3] Chan EH, Sahai V, Conrad C, Brownstein JS (2011). Using Web search query data to monitor dengue epidemics: A new model for neglected tropical disease surveillance. PLoS Negl Trop Dis.

[4] Gubler DJ. Dengue and Dengue Hemorrhagic Fever. Clin. Microbiol. Rev. 1998;11(3):480–96. 10.1128/cmr.11.3.480 PMC88892 9665979

[5] UNDP/World Bank/WHO Special Programme for Research and Training in Tropical Diseases (2007). Report of the Scientific Working Group meeting on dengue Geneva, 1–5 October 2006.

[6] Guzman MG, Halstead SB, Artsob H, Buchy P, Farrar J, Gubler DJ (2010). Dengue: a continuing global threat. Nat Rev Microbiol.

[7] Barbazan P, Yoksan S, Gonzalez JP (2002). Dengue hemorrhagic fever epidemiology in Thailand: description and forecasting of epidemics. Microbes Infect.

[8] Mammen MP, Pimgate C, Koenraadt CJ, Rothman AL, Aldstadt J, Nisalak A (2008). Spatial and temporal clustering of dengue virus transmission in Thai villages. PLoS Med.

[9] Tun-Lin W, Kay BH, Barnes A, Forsyth S (1996). Critical examination of *Aedes aegypti* indices: correlations with abundance. Am J Trop Med Hyg.

[10] Focks DA (2003). A review of entomological sampling methods and indicators for dengue vectors.

[11] Tuksinvaracharn R, Tanayapong P, Pongrattanaman S, Hansasuta P, Bhattarakosol P, Siriyasatien P (2004). Prevalence of dengue virus in *Aedes* mosquitoes during dry season by semi-nested reverse transcriptase-polymerase chain reaction (semi-nested RT-PCR). J Med Assoc Thai.

[12] Thavara U, Siriyasatien P, Tawatsin A, Asavadachanukorn P, Anantapreecha S, Wongwanich R (2006). Double infection of heteroserotypes of dengue viruses in field populations of *Aedes aegypti* and *Aedes albopictus* (Diptera: Culicidae) and serological features of dengue viruses found in patients in southern Thailand. Southeast Asian J Trop Med Public Health.

[13] Chen CF, Shu PY, Teng HJ, Su CL, Wu JW, Wang JH (2010). Screening of dengue virus in field-caught *Aedes aegypti* and *Aedes albopictus* (Diptera: Culicidae) by one-step SYBR green-based reverse transcriptase polymerase chain reaction assay during 2004-2007 in Southern Taiwan. Vector Borne Zoonotic Dis.

[14] Chompoosri J, Thavara U, Tawatsin A, Anantapreecha S, Siriyasatien P (2013). Seasonal monitoring of dengue infection in *Aedes aegypti* and serological feature of patients with suspected dengue in 4 central provinces of Thailand. Thai J Vet Med.

[15] Kesorn K, Ongruk P, Chompoosri J, Phumee A, Thavara U, Tawatsin A (2015). Morbidity rate prediction of dengue hemorrhagic fever (DHF) using the support vector machine and the *Aedes aegypti* infection rate in similar climates and geographical areas. PLoS ONE.

[16] Kittichai V, Montriwat P, Chompoosri J, Bhakdeenuan P, Pengsakul T, Tawatsin A (2015). Relationships between dengue virus infection in mosquito vector, *(Aedes aegypti)*, dengue cases and weather conditions in Samut Sakhon Province, Thailand. Chula Med J.

[17] Earnest A, Tan SB, Wilder-Smith A, Machin D (2012). Comparing statistical models to predict dengue fever notifications. Comput Math Methods Med.

[18] Luz PM, Mendes BVM, Codeço CT, Struchiner CJ, Galvani AP (2008). Time series analysis of dengue incidence in Rio de Janeiro, Brazil. Am J Trop Med Hyg.

[19] Gharbi M, Quenel P, Gustave J, Cassadou S, Ruche GL, Girdary L (2011). Time series analysis of dengue incidence in Guadeloupe, French West Indies: Forecasting models using climate variables as predictors. BMC Infect Dis.

[20] Hii YL, Zhu H, Ng N, Ng LC, Rocklöv J (2012). Forecast of dengue incidence using temperature and rainfall. PLoS Negl Trop Dis.

[21] Choudhury MAH, Banu S, Islam MA (2008). Forecasting dengue incidence in Dhaka, Bangladesh: A time series analysis. Dengue Bull.

[22] Fathima S, Hundewale N. Comparison of classification techniques-SVM and naives bayes to predict the Arboviral disease-Dengue. 2011 IEEE Int. Conf. Bioinforma. Biomed. Workshop BIBMW. 2011. p. 538–9.

[23] Ibrahim F, Faisal T (2010). Non-invasive diagnosis of risk in dengue patients using bioelectrical impedance analysis and artificial neural network. Med Biol Eng Comput.

[24] Lanciotti RS, Calisher CH, Gubler DJ, Chang GJ, Vorndam AV (1992). Rapid detection and typing of dengue viruses from clinical samples by using reverse transcriptase-polymerase chain reaction. J Clin Microbiol.

[25] Chanama S, Anantapreecha S, A-nuegoonpipat A, Sa-gnasang A, Kurane I, Sawanpanyalert P (2004). Analysis of specific IgM responses in secondary dengue virus infections: Levels and positive rates in comparison with primary infections. J Clin Virol Off Publ Pan Am Soc Clin Virol.

[26] Agresti A (2007). An Introduction to Categorical Data Analysis.

[27] Huang X, Williams G, Clements ACA, Hu W (2013). Imported dengue cases, weather variation and autochthonous dengue incidence in cairns, Australia. PLoS ONE.

[28] Burnham K, Anderson D (2002). Model selection and multimodel inference: A practical information-theoretic approach.

[29] Wit E, van den Heuvel E, Romeijn J-W (2012). All models are wrong…:An introduction to model uncertainty. Stat Neerlandica.

[30] Chakravarti I, Laha R, Roy J (1967). Handbook of Methods of Applied Statistics.

[31] Wongkoon S, Jaroensutasinee M, Jaroensutasinee K (2013). Distribution, seasonal variation & dengue transmission prediction in Sisaket, Thailand. Indian J Med Res.

[32] Angel B, Sharma K, Joshi V (2008). Association of ovarian proteins with transovarial transmission of dengue viruses by *Aedes* mosquitoes in Rajasthan, India. Indian J Med Res.

[33] Edillo FE, Sarcos JR, Sayson SL (2015). Natural vertical transmission of dengue viruses in *Aedes aegypti* in selected sites in Cebu City, Philippines. J Vector Ecol.

[34] Strickman D, Kittayapong P (2003). Dengue and Its Vectors in Thailand: Calculated Transmission Risk from Total Pupal Counts of *Aedes aegypti* and Association of Wing-Length Measurements with Aspects of the Larval Habitat. Am J Trop Med Hyg.

[35] Sanchez L, Cortinas J, Pelaez O, Gutierrez H, Concepción D, Van der Stuyft P (2010). Breteau Index threshold levels indicating risk for dengue transmission in areas with low *Aedes* infestation. Trop Med Int Health TM IH.

[36] Bowman LR, Runge-Ranzinger S, McCall PJ (2014). Assessing the Relationship between Vector Indices and Dengue Transmission: A Systematic Review of the Evidence. PLoS Negl Trop Dis.

[37] Hu SF, Li M, Zhong LL, Lu SM, Liu ZX, Pu JY (2015). Development of reverse-transcription loop-mediated isothermal amplification assay for rapid detection and differentiation of dengue virus serotypes 1-4. BMC Microbiol.

[38] Sriworarat C, Phumee A, Mungthin M, Leelayoova S, Siriyasatien P (2015). Development of loop-mediated isothermal amplification (LAMP) for simple detection of *Leishmania* infection. Parasit Vectors.

